# Nursing and allied health workforce in Australian public rheumatology departments is inadequate: a cross-sectional observational study

**DOI:** 10.1007/s00296-024-05547-y

**Published:** 2024-03-16

**Authors:** Glen A. Whittaker, Catherine L. Hill, Linda A Bradbury, Janet R. Millner, Harrison Cliffe, Daniel R. Bonanno, Sia Kazantzis, Hylton B. Menz

**Affiliations:** 1 Discipline of Podiatry, School of Allied Health, Human Services and Sport, La Trobe University, Melbourne, VIC Australia; 2 Rheumatology Unit, The Queen Elizabeth Hospital, Woodville, SA Australia; 3 Rheumatology Unit, Royal Adelaide Hospital, Adelaide, SA Australia; 4 Discipline of Medicine, University of Adelaide, Adelaide, SA Australia; 5 Department of Rheumatology, Gold Coast University Hospital, 1 Hospital Boulevard, Southport, QLD Australia; 6 Menzies Institute for Medical Research, University of Tasmania, Hobart, TAS Australia; 7 Pharmacy Department, Townsville Hospital and Health Service, 100 Angus Smith Drive, Douglas, QLD Australia; 8 La Trobe Sports and Exercise Medicine Research Centre, School of Allied Health, Human Services and Sport, La Trobe University, Melbourne, VIC Australia

**Keywords:** Health workforce, Rheumatology, Allied health personnel, Nursing staff, Surveys and questionnaires

## Abstract

Rheumatological conditions are complex and impact many facets of daily life. Management of people with rheumatological conditions can be optimised through multidisciplinary care. However, the current access to nursing and allied health professionals in Australia is unknown. A cross-sectional study of nursing and allied health professionals in Australian public rheumatology departments for adult and paediatric services was conducted. The heads of Australian public rheumatology departments were invited to report the health professionals working within their departments, referral pathways, and barriers to greater multidisciplinary care. A total of 27/39 (69.2%) of the hospitals responded. The most common health professionals *within* departments were nurses (*n* = 23; 85.2%) and physiotherapists (*n* = 10; 37.0%), followed by pharmacists (*n* = 5; 18.5%), psychologists (*n* = 4; 14.8%), and occupational therapists (*n* = 4; 14.8%). No podiatrists were employed within departments. Referral pathways were most common for physiotherapy (*n* = 20; 74.1%), followed by occupational therapy (*n* = 15; 55.5%), podiatry (*n* = 13; 48.1%), and psychology (*n* = 6; 22%). The mean full-time equivalent of nursing and allied health professionals per 100,000 population in Australia was 0.29. Funding was identified as the most common barrier. In Australia, publicly funded multidisciplinary care from nurses and allied health professionals in rheumatology departments is approximately 1.5 days per week on average. This level of multidisciplinary care is unlikely to meet the needs of rheumatology patients. Research is needed to determine the minimum staffing requirements of nursing and allied health professionals to provide optimal care.

**Supplementary Information:**

The online version contains supplementary material available at 10.1007/s00296-024-05547-y.

## Introduction

Multidisciplinary care has been shown to improve patient outcomes, which is particularly important for people with rheumatological conditions that affect multiple body systems, body regions, and tissue types [1]. In addition, these conditions impact several facets of life such as work, sleep, diet, sexual function, and sport and exercise [2–5]. Guidelines recommend that optimal management of patients with rheumatological conditions should include multidisciplinary care, including contributions from nursing and allied health professionals [6–8]. Indeed, Australia’s Health Minister in 2023 outlined that the second of three foundations for healthcare reform in Australia is to enable more multidisciplinary and team-based approaches to healthcare [9]. Despite this, it is unclear whether patients in Australia with rheumatological conditions have access to publicly funded multidisciplinary care.

In the United Kingdom (UK), a 2017 audit of staffing within rheumatology teams found that almost all had access to a specialist nurse; however, access to occupational therapists (75% of rheumatology teams), physiotherapists (73% of rheumatology teams), and podiatrists (48% of rheumatology teams) was below the national guidance for multidisciplinary care [10]. The number of health professionals working within rheumatology teams appears to have declined, as indicated by a 2021 report from the British Society of Rheumatology [11]. This audit examined multidisciplinary staff within rheumatology teams, and the referral pathways to services within the Trust (i.e. the organisation). Among rheumatology teams, 50% had no access to physiotherapists, while 52% of patients lacked access to occupational therapists, 80% had no access to podiatrists, and 82% had no access to psychologists. Regarding Trusts, 5% did not have the ability to refer for occupational therapy services, 7% for physiotherapy services, 24% for podiatry services, and 62% for psychology services. These data suggest that most patients have access to nursing and physiotherapy either within the rheumatology team or as a referral within the organisation. However, access to other allied health professionals such as podiatrists, occupational therapists, and psychologists is more difficult. Limited access to nursing and allied health professionals also occurs in other regions including Southeast Asia, the Asia–Pacific [12], and Arab countries [13].

Understanding whether patients with rheumatological conditions can access publicly funded multidisciplinary care is important for several reasons. Multidisciplinary care improves patient outcomes, yet people with lower incomes may not be able to afford privately funded multidisciplinary care, potentially exacerbating their disadvantage [14]. Therefore, we need to understand the level of publicly funded multidisciplinary care to know whether this meets the needs of the community. This study can also help hospitals identify gaps in referral pathways, leading to future research to address these gaps, fund new nursing and allied health positions, and assist rheumatologists to provide optimal care for their patients. Accordingly, the aim of this study was to determine patient access to publicly funded multidisciplinary care from nursing and allied health professionals within Australian public tertiary rheumatology departments.

## Methods

This observational, cross-sectional study used an online survey to collect data about nursing and allied health professionals working in public rheumatology departments in Australia and perceived barriers to greater multidisciplinary care. To develop survey questions, an iterative process was used that involved three steps: (i) a literature search to identify past research about nursing and allied health professionals working in rheumatology and possible barriers to multidisciplinary care, (ii) development of survey questions by the lead author, and (iii) refinement of survey questions in collaboration with relevant health professionals working in rheumatology including a rheumatologist, a nurse practitioner, a physiotherapist, podiatrists, and a pharmacist. This study is reported in accordance with the Strengthening the Reporting of Observational Studies in Epidemiology (STROBE) checklist [15]. Ethics approval was obtained from the La Trobe University Human Ethics Committee (HEC21432) and informed consent was obtained from all individual participants included in the study.

### Participants

A list of all public rheumatology departments in Australia obtained from the Australian Rheumatology Association (*N* = 39). An a priori sample size calculation was not performed given that we know the total population. Invitations to complete a survey were emailed to the heads/leads of each rheumatology department to complete on behalf of their respective department. Email invitations were initially sent in February 2022 and the survey was closed in August 2022.

### Outcomes

Data were collected using an online survey (REDCap, Vanderbilt University, Nashville, USA). Participants were asked to respond to items about the absolute number, full-time equivalent, funding source, and barriers to greater involvement of nursing and allied health professionals in their department. Participants were also asked items about referral pathways to nursing and allied health professionals within their organisation. Responses to items were provided using either a yes/no format, free text, or selection of perceived barriers to greater multidisciplinary care from a pre-determined list. The survey questions are available in Online Resource 1.

### Statistical analysis

Data were reported descriptively for responses to the number of nursing and allied health professionals in each department and the perceived barriers to multidisciplinary care. To determine the full-time equivalent of nursing and allied health professionals per primary health network, each hospital was mapped to a primary health network using the ‘MyHospitals mapping details’ list from the Australian Institute of Health and Welfare [16]. Population data were obtained for each primary health network using the ‘Social Health Atlas of Australia’ produced by the Public Health Information Development Unit, Torrens University, Australia [17].

## Results

Invitations were emailed to the heads of 39 public rheumatology departments, and 27 responded, yielding a response rate of 69.2%. Table 1 displays the total number of adult rheumatology services that have each profession working in their department, plus the mean, full-time equivalent, and funding source of each profession. In addition, this table displays whether a referral pathway existed for each profession within the hospital. The rheumatologist workforce consisted of staff specialists in all departments (mean 6.1 per department) and 17/28 departments (60.7%) included visiting medical officers. For nurses, 19/27 (70.3%) hospitals employed registered nurses, while 5/27 (18.5%) employed rheumatology nurse practitioners. Specialist clinics run by rheumatology departments (e.g. scleroderma clinics) included multidisciplinary care involving nurses in 15/24 (62.5%) hospitals, physiotherapists in 12/27 (44.4%), psychologists in 4/11 (36.4%), occupational therapists in 12/18 (66.7%), and pharmacists in 5/5 (100.0%).

**Table 1:** Rheumatologists, nurses, and allied health professionals working in Australian public rheumatology departments

	Rheumatologists	Nurses	Physiotherapists	Podiatrists	Psychologists	Occupational therapists	Pharmacists
Health professionals working within rheumatology departments							
Within the department, *n* (%)	27/27 (100.0)	23/27 (85.1)	10/27 (37.0)	0 (0.0)	4/27 (14.8)	4/27 (14.8)	5/27 (18.5)
Mean staff (SD)	6.1 (2.5)	2.0 (1.1)	2.0 (1.6)	–	1.0 (0.0)	1.0 (0.0)	1.0 (0.0)
Mean full-time equivalent (SD)	2.6 (1.3)	1.4 (0.8)	1.2 (1.7)	–	0.6 (0.3)	0.3 (0.1)	0.1 (0.1)
Funded from hospital, *n* (%)	–	21/23 (91.3)	10/10 (100)	–	4/4 (100.0)	4/4 (100.0)	5/5 (100.0)
Referral pathways to health professionals in the hospital but outside rheumatology departments							
Outside the department, *n* (%)	–	2/27 (7.4)	20/27 (74.1)	13/27 (48.1)	6/27 (22.2)	15/27 (55.5)	*
Funded from hospital, *n* (%)	–	1/2 (50.0)	18/20 (90.0)	13/13 (100.0)	6/6 (100.0)	15/15 (100.0)	*

^*^It was assumed that public hospitals would all have a pharmacy department within the organisation

### Barriers

Table 2 displays the perceived barriers to greater multidisciplinary care *within* adult rheumatology services. Funding was identified as the most common barrier for each profession, while limited resources (e.g. time, staff, etc.) to create a position and limited space/facilities were also common barriers. Table 3 displays the perceived barriers to greater multidisciplinary care *outside* rheumatology departments, but within the same organisation. The most common barrier was no referral pathway within the organisation, with funding being the second most common barrier.

**Table 2:** Perceived barriers to multidisciplinary health professionals working *within* rheumatology departments

	Nurses	Physiotherapists	Podiatrists	Psychologists	Occupational therapists	Pharmacists
Funding, *n* (%)	4/27 (14.8)	17/27 (62.9)	22/27 (81.4)	20/27 (74.1)	18/27 (66.7)	13/27 (48.1)
Lack of clinical need, *n* (%)	0/27 (0.0)	0/27 (0.0)	6/27 (22.2)	4/27 (14.8)	3/27 (11.1)	8/27 (29.6)
Limited resources to create position, *n* (%)	1/27 (3.7)	4/27 (14.8)	5/27 (18.5)	6/27 (22.2)	5/27 (18.5)	3/27 (11.1)
Limited health professionals with sufficient skills/knowledge, *n* (%)	2/27 (7.4)	0/27 (0.0)	3/27 (11.1)	3/27 (11.1)	0/27 (0.0)	0/27 (0.0)
Access to health professionals external to the department, *n* (%)	0/27 (0.0)	2/27 (7.4)	6/27 (22.2)	1/27 (3.7)	5/27 (18.5)	6/27 (22.2)
Limited space/facilities, *n* (%)	0/27 (0.0)	5/27 (18.5)	5/27 (18.5)	6/27 (22.2)	6/27 (22.2)	5/27 (18.5)
Other, *n* (%)	0/27 (0.0)	1/27 (3.7)	1/27 (3.7)	0/27 (0.0)	0/27 (0.0)	2/27 (7.4)

**Table 3:** Perceived barriers to multidisciplinary care *outside* rheumatology departments

	Nurses	Physiotherapists	Podiatrists	Psychologists	Occupational therapists	Pharmacists
Health professional within the department	15/27 (55.5)	1/27 (3.7)	0/27 (0.0)	2/27 (7.4)	3/27 (11.1)	*
Funding	6/27 (22.2)	1/27 (3.7)	4/27 (14.8)	11/27 (40.7)	6/27 (22.2)	*
Lack of clinical need	2/27 (7.4)	0/27 (0.0)	3/27 (11.1)	0/27 (0.0)	0/27 (0.0)	*
Limited health professionals with sufficient skills/knowledge	5/27 (18.5)	1/27 (3.7)	1/27 (3.7)	2/27 (7.4)	1/27 (3.7)	*
Other	3/27 (11.1)	1/27 (3.7)	1/27 (3.7)	3/27 (11.1)	1/27 (3.7)	*
No referral pathway within organisation	11/27 (40.7)	4/27 (14.8)	9/27 (33.3)	14/27 (51.8)	5/27 (18.5)	*
Too busy to see our patients	3/27 (11.1)	1/27 (3.7)	3/27 (11.1)	3/27 (11.1)	2/27 (7.4)	*

^*^It was assumed that referring to the pharmacy department outside of rheumatology would be common practice

### Paediatric services

A total of 10/27 hospitals (37.0%) reported offering a service for paediatric patients, and 7/10 (70.0%) reported that nursing and allied health professionals provided multidisciplinary care. There were 5/10 (50.0%) rheumatology departments that reported having referral pathways to nursing and allied health professionals outside the rheumatology department but within the same organisation. Table 4 displays the total number of each profession working in their department, plus the mean full-time equivalent. Because there were few paediatric services without nursing and allied health professionals, the barriers to multidisciplinary care data were minimal. Themes were similar for adult services and responses mainly focussed on a lack of funding, a lack of health professionals with sufficient clinical skills, and no referral pathways within the organisation.

**Table 4:** Nurses and allied health professionals working in paediatric services in Australian public rheumatology departments

	Nurses	Physiotherapists	Podiatrists	Occupational therapists	Psychologists	Pharmacists
Within department, *n* (%)	7/10 (70.0)	4/10 (40.0)	0/10 (0.0)	3/10 (30.0)	2/10 (20.0)	2/10 (20.0)
Mean full-time equivalent	0.6	0.3	0.0	0.2	0.3	0.1
Referral pathways to health professionals outside the department, *n* (%)	1/5 (20.0)	4/5 (80.0)	0/0 (0.0)	3/5 (60.0)	2/5 (40.0)	*

^*^It was assumed that referring to the pharmacy department outside of rheumatology would be common practice

### Multidisciplinary care by population

Table 5 displays the full-time equivalent of nursing and allied health professionals by primary health network, population per primary health network, and self-reported population with arthritis per primary health network. The mean full-time equivalent of all nursing and allied health professionals per 100,000 population in Australia was 0.29, and the total full-time equivalent from hospitals participating in this study was 46.2.

**Table 5:** Full-time equivalent of nursing and allied health professionals by primary health network, population per primary health network, and self-reported population with arthritis per primary health network

Primary health network	Nurses	Physiotherapists	Podiatrists	Psychologists	Occupational therapists	Pharmacists	Total FTE	Estimated population	FTE per 100,000 estimated population	Population with arthritis
Queensland										
Northern Queensland	1.0	0.0	0.0	0.0	0.0	0.0	1.0	703,393	0.14	56,465
Brisbane South	3.3	0.2	0.0	0.0	0.2	0.1	3.8	1,192,099	0.32	89,655
Brisbane North	1.8	0.0	0.0	0.0	0.0	0.1	1.9	1,045,037	0.18	85,387
Gold Coast	1.8	0.0	0.0	0.0	0.0	0.2	2.0	640,687	0.31	53,462
New South Wales										
Hunter New England and Central Coast	2.6	0.0	0.0	0.0	0.0	0.0	2.6	1,304,879	0.19	149,878
Northern Sydney	0.8	1.5	0.0	0.0	0.5	0.0	2.8	922,281	0.30	57,210
Central and Eastern Sydney	4.0	5.5	0.0	0.0	0.2	0.0	9.7	1,542,456	0.62	92,299
South-Western Sydney	1.7	0.8	0.0	0.0	0.0	0.0	2.5	1,059,075	0.23	77,771
Australian Capital Territory										
Australian Capital Territory	2.0	0.2	0.0	0.0	0.0	0.0	2.2	454,499	0.48	35,106
Victoria										
North-Western Melbourne	3.4	0.0	0.0	0.0	0.0	0.0	3.4	1,825,198	0.18	110,429
Eastern Melbourne	1.7	0.0	0.0	0.0	0.0	0.1	1.8	1,519,971	0.11	116,333
Tasmania										
Tasmania	1.3	0.8	0.0	0.0	0.0	0.0	2.1	557,571	0.37	68,079
South Australia										
Adelaide	3.2	0.7	0.0	0.8	0.0	0.0	4.7	1,269,482	0.37	120,005
Western Australia										
Perth South	3.0	0.0	0.0	0.0	0.0	0.2	3.2	1,028,929	0.31	78,866
Perth North	1.8	1.0	0.0	0.5	0.2	0.0	3.5	1,094,901	0.32	78,481

*FTE* full-time equivalent

## Discussion

We determined the number of nursing and allied health professionals employed in Australian public rheumatology departments to establish the current availability of multidisciplinary care by nursing and allied health professionals for patients with rheumatological conditions, and identified potential barriers to greater access to nursing and allied health professionals in these rheumatology departments. We found that publicly funded multidisciplinary care is relatively low compared to other countries such as the UK and Canada [11, 18, 19]. On average, nursing and allied health professionals in Australia provide an equivalent of 1.5 days per week of care in Australian rheumatology departments. There were consistent barriers to greater involvement of nursing and allied health professionals, which were mostly administrative (i.e. funding, referral pathways) rather than those related to clinical practice.

We found that, on average, multidisciplinary care in adult services from nurses and allied health professionals accounted for approximately 0.3 full-time equivalent staff per 100,000 population. This can be compared to a study from the UK that found physiotherapists alone comprised a mean full-time equivalent of 0.3 per 100,000 population [19]. There is no recommendation for the minimum full-time equivalent of allied health professionals required to support the rheumatology population. Australian guidelines for rheumatologists suggest a full-time equivalent of 1.0 per 50,000 population [20], which is similar to the British Society for Rheumatology who recommend 1.0 rheumatologists per 60,000 population. For nurses, the British Society for Rheumatology recommend a ratio of 1 specialist rheumatology nurse per rheumatologist, which would imply a full-time equivalent of 1.0 specialist rheumatology nurse per 60,000 population [11]. If this guidance was applied to Australia, most regions as outlined in Table 4 would be below this standard. For example, the highest full-time equivalent for nurses in a primary health network can be estimated at 0.27 per 100,000 in the *Brisbane South* Primary Heath Network (Table 4). Development of minimum staffing levels required in rheumatology is needed so rheumatology departments can understand minimum staffing requirements for optimal multidisciplinary care.

Nurses were the most common non-physician health professional involved in multidisciplinary care within adult rheumatology departments. In 85% of rheumatology departments, an average of two nurses worked a mean full-time equivalent of 1.4. Physiotherapists represented the second most prevalent health profession within these departments (37%), with an average of 2.0 physiotherapists working a mean full-time equivalent of 1.2. The percentage of departments with a physiotherapist working within the department is much lower than 2021 data from the UK, which suggests that 50% of departments have a physiotherapist within their team [11]. Patients in Australian rheumatology departments have similar access to psychologists, occupational therapists and pharmacists, ranging between 14 and 18%. These figures are all below recent UK data, which report that patients in the UK have access to psychologists in 18% of Trusts, occupational therapists in 48%, and pharmacists in 48% [11]. Patients do not have access to any podiatrists within public rheumatology departments in Australia, which compares to 20% in the UK [11]. This is surprising, given that feet are commonly affected in people with rheumatological conditions [21–23], and foot care is one of the most common additional care needs requested by people with rheumatoid arthritis [24].

While not all Australian rheumatology departments have embedded nursing and allied health professionals, the hospital generally has health professionals that can manage rheumatology patients. These health professionals can provide multidisciplinary care, although they may not have the specialised knowledge required to optimally manage rheumatology patients. Patient referral pathways to allied health professionals external to departments are more common than those within the department. However, Australian hospitals with referral pathways are lower than the UK for physiotherapy (74% of Australian hospitals compared to 93% in the UK) and podiatry (48% of Australian hospitals compared to 76% in the UK) [11]. The number of hospitals with referral pathways was similar between Australia and the UK for occupational therapy and psychology. We have not examined referral pathways for nursing and pharmacy, given that referrals are unlikely for nursing and we assumed that most hospitals have a pharmacy department.

Paediatric services were provided in a public setting by 37% of hospitals. The majority of paediatric services included multidisciplinary care (70%), which is necessary given the complex needs of paediatric patients. Similar to adult services, nurses (70%) and physiotherapists (40%) were the most common health professionals working within rheumatology departments, while occupational therapists (30%), psychologists (20%), and pharmacists (20%) were also included in some departments. The full-time equivalent of staff in these departments was lower than in adult departments, ranging between 0.0 (for podiatrists) and 0.6 (for nurses). These staffing levels are lower than those reported in Canada, but similar to levels reported in Southeast Asia and the Asia–Pacific regions with the exception of nurses. In Canada, 100% of paediatric rheumatology centres have nurses, 80% have physiotherapists, and 60% have occupational therapists [18]. However, in Southeast Asia and the Asia–Pacific, only 15% of paediatric multidisciplinary teams have rheumatology nurses, 31% have occupational therapists, and 49% have physiotherapists [12].

Barriers to multidisciplinary care within rheumatology departments are funding, limited space/facilities, and limited resources (e.g. time) to create a position. A 2017 report into rheumatology nursing in Australia also identified funding as the most common barrier to nurses contributing to their full potential [25]. Barriers to patients accessing multidisciplinary care outside rheumatology departments but within the same organisation were primarily a lack of funding, which is similar to the barriers within departments. However, the second most common barrier was a lack of a referral pathway to the service within the organisation, followed by the service being too busy to accept rheumatology patients. Overcoming these barriers, although challenging, will likely result in improvements to patient care given a multidisciplinary approach may improve patient outcomes. Further research will need to evaluate the clinical and cost-effectiveness of additional nursing and allied health staff on patient outcomes.

When interpreting the conclusions from this study there are some limitations that should be taken into consideration. Unfortunately, not all rheumatology departments responded to the invitation, and we may not have sent invitations to all departments, so these data may not represent the entire Australian workforce. We are unclear why rheumatology departments decided not to participate, but a possible reason may include a lack of time to complete the survey. We have presented most data as means across hospitals rather than totals to present the findings in the most meaningful manner. We also did not include all allied health professionals in our survey, and there may be other health professionals who may work in rheumatology departments or be referred to within the organisation that we have not included. Finally, nursing and allied health staff per population were estimates based on each hospitals associated primary health network. The true catchment population of each hospital, and the associated nursing and allied health required to service that population may differ in some regions.

Several areas for future research have been identified in this study. There is a need for ongoing research to evaluate the staffing levels of nursing and allied health professionals working in rheumatology over time. These data can inform areas of need and provide important data to compare to other regions and any identified benchmarks. Workforce analysis surveys are needed to determine the minimum nursing and allied health professionals required in an Australian rheumatology setting. We also highlighted funding and the lack of referral pathways within public rheumatology hospitals as key barriers. Future research can explore the clinical and cost-effectiveness of greater involvement of nursing and allied health professionals in rheumatology, as well as strategies to improve referral pathways.

In conclusion, access to multidisciplinary care can improve outcomes, especially for people with rheumatological conditions that affect multiple bodily systems and facets of life. Furthermore, accessing publicly funded multidisciplinary care is important for people who are financially vulnerable. Rheumatology departments in Australia include multidisciplinary care for adults and paediatric patients that mostly involves nurses and physiotherapists, however staffing levels in Australia were much lower than in countries with health systems comparable to Australia, which is unlikely to provide optimal care. Similarly, other allied health professionals such as occupational therapists, psychologists and pharmacists were included within some rheumatology departments, but this was at a much lower level than in the UK and was not evenly distributed across Australia. There were no podiatrists working within rheumatology departments in Australia, which is a notable difference to podiatry access in the UK. Patients have access to multidisciplinary care through referral to other services, but again this occurs at a much lower level than the UK, and a lack of referral pathways to these services are a key barrier along with a lack of funding.

### Supplementary Information

Below is the link to the electronic supplementary material.

Supplementary file1 (PDF 218 KB)

## Data Availability

Data are available from the authors upon reasonable request.
