# Supplementary material for: Nursing and allied health workforce in Australian public rheumatology departments is inadequate: a cross-sectional observational study
Source: Rheumatol Int. 2024 Mar 16;44(5):901–8. doi: 10.1007/s00296-024-05547-y (PMC10980610; doi:10.1007/s00296-024-05547-y)
Supplement: Supplementary file 1 — Supplementary file1 (PDF 218 KB) [file 296_2024_5547_MOESM1_ESM.pdf]

Journal
Rheumatology International

Article title

Nursing and allied health workforce in Australian public rheumatology departments is inadequate: a cross-sectional observational study

Authors
Glen A Whittaker (ORCID 0000-0002-2102-7777)[1], Catherine L Hill (0000-0001-8289-4922)[2 3 4], Linda Bradbury[5], Janet R Millner[6], Harrison Cliffe[7], Daniel R Bonanno (ORCID 0000-0002-0825-0608)[1], Sia Kazantzis[1], Hylton B Menz (ORCID 0000-0002-2045-3846)[1 8]

Affiliations
[1] Discipline of Podiatry, School of Allied Health Human Services and Sport, La Trobe University, Melbourne, Victoria, Australia
[2] Rheumatology Unit, The Queen Elizabeth Hospital, Woodville, South Australia, Australia
[3] Rheumatology Unit, Royal Adelaide Hospital, Adelaide, South Australia, Australia
[4] Discipline of Medicine, University of Adelaide, Adelaide, South Australia, Australia
[5] Department of Rheumatology, Gold Coast University Hospital, 1 Hospital Boulevard, Southport, Queensland, Australia
[6] Menzies Institute for Medical Research, University of Tasmania, Hobart, Tasmania, Australia
[7] Pharmacy Department, Townsville Hospital and Health Service, 100 Angus Smith Drive, Douglas, Queensland, Australia
[8] La Trobe Sports and Exercise Medicine Research Centre, School of Allied Health Human Services and Sport, La Trobe University, Melbourne, Victoria, Australia

Corresponding author
Dr Glen A Whittaker, g.whittaker@latrobe.edu.au

Nursing and allied health professionals in Australian public rheumatology departments

The research is being carried out by the following researchers:

Role

Name

Organisation

Chief investigator

Associate investigators

Dr Glen Whittaker

Professor Hylton Menz

Dr Daniel Bonanno

Ms Sia Kazantzis

Professor Catherine Hill

Ms Linda Bradbury

Ms Janet Millner

Mr Harrison Cliffe

La Trobe University

La Trobe University

La Trobe University

La Trobe University

University of South Australia

Gold Coast University Hospital

University of Tasmania

Townsville University Hospital

Research funder

This research has received $4,950.00 from the School of Allied Health, Human Services and Sport, La Trobe University.

1. What is the study about?

You are invited to participate in a study of the nursing and allied health staff who work in public rheumatology departments in Australia. We hope to learn about the access patients have to nursing and allied health professionals, and if there are geographic differences in access.

Your contact details were obtained from the Australian Rheumatology Association.

2. Do I have to participate?

Being part of this study is voluntary. If you want to be part of the study, we ask that you read the information below carefully.

You can read the information below and decide at the end if you do not want to participate. If you decide not to participate this won't affect your relationship with La Trobe University or any other listed organisation.

3. Who is being asked to participate?

You have been asked to participate because you are a Lead or Manager of an Australian public rheumatology department.

4. What will I be asked to do?

If you want to take part in this study, we will ask you to complete an online survey. It will take less than 10 minutes of your time to be part of this study. We would like you to answer on behalf of the staff in your rheumatology department.

5. What are the benefits?

While there are no direct benefits for you taking part in this study, the expected benefits to society in general are that we will learn more about the provision of care in public rheumatology departments nursing and allied health professionals. This can help to guide future staffing decisions to provide optimum care for patients.

6. What are the risks?

With any study there are (1) risks we know about, (2) risks we don't know about, and (3) risks we don't expect. If you experience something that you aren't sure about, please contact us immediately so we can discuss the best way to manage your concerns.

Name/Organisation

Position

Telephone

Email

Dr Glen Whittaker, La Trobe University

Lecturer

(03) 9479 5785

g.whittaker@latrobe.edu.au

We do not foresee any risks associated with this study.

7. What will happen to information about me?

By clicking on the ‘I agree, start questionnaire’ button, this tells us you want to take part in the study.

- We will collect information about you in ways that will reveal who you are.

- We will store information about you in ways that will reveal who you are.

- We will publish information about you in ways that will not be identified in any type of publication from this study.

- We will keep your information for 5 years after the project is completed. After this time we will destroy all of your data.

- The storage, transfer and destruction of your data will be undertaken in accordance with the Research Data Management Policy https://policies.latrobe.edu.au/document/view.php?id=106/.

- The personal information you provide will be handled in accordance with applicable privacy laws, any health information collected will be handled in accordance with the Health Records Act 2001 (Vic). Subject to any exceptions in relevant laws, you have the right to access and correct your personal information by contacting the research team.

8. Will I hear about the results of the study?

We will let you know about the results of the study by email once the findings are published.

9. What if I change my mind?

If you no longer want to complete the questionnaire, simply close the web browser. If you change your mind after clicking on the ‘Submit’ button, we can withdraw your responses because we can link who you are with your questionnaire responses.

Your decision to withdraw at any point will not affect your relationship with La Trobe University or any other organisation listed.

10. Who can I contact for questions or want more information?

If you would like to speak to us, please use the contact details below:

Name/Organisation

Position

Telephone

Email

Dr Glen Whittaker, La Trobe University

Lecturer

(03) 9479 5785

g.whittaker@latrobe.edu.au

11. What if I have a complaint?

If you have a complaint about any part of this study, please contact:

Ethics Reference Number

Position

Telephone

Email

HEC21432

Senior Research Ethics Officer

+61 3 9479 1443

humanethics@latrobe.edu.au

Consent

Consent Form - Declaration by Participant

I (the participant) have read and understood the Participant Information Statement, and any questions have been answered to my satisfaction. I agree to participate in the study, I know I can withdraw at any time until [four weeks] following the collection of my data. I agree that information provided by me or with my permission during the project may be included in a thesis, presentation and published in journals on the condition that I cannot be identified.

By clicking "Next" I provide consent and agree to participate in the study.

I would like my information collected for this research study to be:	◯ Used for this and future closely related studies<br>◯ Used for this study only

Rheumatologists

At which hospital are you the Head/Manager of the rheumatology department?

◯ My hospital isn't listed
◯ State Forensic Mental Health Service
◯ Justice Health Services
◯ The Children's Hospital at Westmead
◯ Sydney Children's Hospital
◯ Sacred Heart Health Service
◯ St Vincent's Hospital [Darlinghurst]
◯ St Joseph's Hospital
◯ Balmain Hospital
◯ Canterbury Hospital
◯ Royal Prince Alfred Hospital
◯ Tresillian Care Centres
◯ Concord Repatriation Hospital
◯ Royal Prince Alfred Institute of Rheumatology & Orthopaedics
◯ Karitane
◯ Camden Hospital
◯ Fairfield Hospital
◯ Liverpool Hospital
◯ Campbelltown Hospital
◯ Bankstown Lidcombe Hospital
◯ Braeside Hospital
◯ Bowral Hospital
◯ Sydney Hospital / Sydney Eye Hospital
◯ Calvary Health Care - Sydney
◯ Gower Wilson Multi Purpose Service
◯ War Memorial Hospital
◯ Prince of Wales Hospital
◯ St George Hospital NSW
◯ Sutherland Hospital
◯ Royal Hospital for Women
◯ Bulli Hospital
◯ Coledale Hospital
◯ Kiama Hospital
◯ Milton Ulladulla Hospital
◯ Port Kembla Hospital
◯ Shoalhaven Hospital
◯ Wollongong Hospital
◯ Shellharbour Hospital
◯ Illawarra Mental Health Services
◯ David Berry Hospital
◯ Auburn Hospital
◯ Blacktown Hospital
◯ Mount Druitt Hospital
◯ Westmead Hospital
◯ Blue Mountains Hospital
◯ Nepean Hospital
◯ Springwood Hospital
◯ Hawkesbury Hospital
◯ Tresillian Family Care Centre, Kingswood
◯ Wentworth Psychiatric Services
◯ Lithgow Hospital
◯ Portland Tabulam Health Centre
◯ Greenwich Hospital
◯ Neringah Hospital
◯ Hornsby Ku-ring-gai Hospital
◯ Manly Hospital
◯ Mona Vale Hospital
◯ Royal North Shore Hospital
◯ Royal Rehabilitation Hospital
◯ Ryde Hospital
◯ Gosford Hospital
◯ Woy Woy Hospital
◯ Long Jetty Health Care Centre
◯ Wyong Hospital
◯ Armidale Hospital
◯ Barraba Multi Purpose Service
◯ Bingara Multi Purpose Service
◯ Boggabri Multi Purpose Service

◯ Glen Innes Hospital
◯ Gunnedah Hospital
◯ Guyra Multi Purpose Service
◯ Inverell Hospital
◯ Manilla Hospital
◯ Moree Hospital
◯ Narrabri Hospital
◯ Tenterfield Hospital
◯ Quirindi Hospital
◯ Tamworth Hospital
◯ Tingha Multi Purpose Service
◯ Vegetable Creek Multi Purpose Service Emmaville
◯ Walcha Multi Purpose Service
◯ Warialda Multi Purpose Service
◯ Wee Waa Hospital
◯ Werris Creek Hospital
◯ Bulahdelah Hospital
◯ Gloucester Soldiers' Memorial Hospital
◯ Manning Hospital
◯ Wingham Hospital
◯ Cessnock Hospital
◯ Dungog Hospital
◯ Kurri Kurri Hospital
◯ Maitland Hospital
◯ Merriwa Multi Purpose Service
◯ Muswellbrook Hospital
◯ Denman Multi Purpose Service
◯ Calvary Mater Newcastle
◯ Belmont Hospital
◯ Scott Memorial Hospital, Scone
◯ Singleton Hospital
◯ Wilson Memorial Community Hospital, Murrurundi
◯ Tomaree Community Hospital
◯ John Hunter Hospital
◯ Ballina District Hospital
◯ Byron Bay Hospital
◯ Coraki Hospital
◯ Casino and District Memorial Hospital
◯ Bonalbo Hospital
◯ Grafton Base Hospital
◯ Kyogle Multi Purpose Service
◯ Lismore Base Hospital
◯ Nimbin Multi Purpose Service
◯ Maclean District Hospital
◯ Mullumbimby Hospital
◯ Murwillumbah District Hospital
◯ The Tweed Hospital
◯ Urbenville Multi Purpose Service
◯ Riverlands Drug and Alcohol Centre
◯ Bellinger River District Hospital
◯ Coffs Harbour Hospital
◯ Dorrigo Multi Purpose Service
◯ Kempsey District Hospital
◯ Macksville District Hospital
◯ Wauchope District Memorial Hospital
◯ Port Macquarie Base Hospital
◯ Batemans Bay Hospital
◯ South East Regional Hospital
◯ Bombala Multi Purpose Service
◯ Braidwood Multi Purpose Service
◯ Cooma Hospital and Health Service
◯ Crookwell District Hospital
◯ Delegate Multi Purpose Service
◯ Goulburn Base Hospital
◯ Moruya Hospital
◯ Pambula Hospital
◯ Queanbeyan Hospital
◯ Bourke Street Health Service
◯ Yass District Hospital
◯ Lake Cargelligo Multi Purpose Service
◯ Wyalong Hospital

◯ Barham Hospital
◯ Berrigan Multi Purpose Service
◯ Culcairn Multi Purpose Service
◯ Corowa Health Service
◯ Deniliquin Hospital
◯ Finley Hospital
◯ Henty Multi Purpose Service
◯ Holbrook Hospital
◯ Jerilderie Multi Purpose Service
◯ Mercy Care Hospital - Albury
◯ Urana Multi Purpose Service
◯ Tocumwal Hospital
◯ Tumbarumba Multi Purpose Service
◯ Boorowa Multi Purpose Service
◯ Mercy Care Hospital - Young
◯ Murrumburrah-Harden Hospital
◯ Young Hospital
◯ Batlow/Adelong Multi Purpose Service
◯ Griffith Base Hospital
◯ Gundagai Hospital
◯ Hay Hospital
◯ Hillston Hospital
◯ Junee Multi Purpose Service
◯ Coolamon-Ganmain Multi Purpose Service
◯ Leeton Hospital
◯ Lockhart Hospital
◯ Narrandera Hospital
◯ Temora Hospital
◯ Tumut Hospital
◯ Wagga Wagga Hospital
◯ Cootamundra Hospital
◯ Bourke Multi Purpose Service
◯ Brewarrina Multi Purpose Service
◯ Cobar Health Service
◯ Collarenebri Multi Purpose Service
◯ Coolah Multi Purpose Service
◯ Coonabarabran Health Service
◯ Baradine Multi Purpose Service
◯ Coonamble Multi Purpose Service
◯ Gulargambone Multi Purpose Service
◯ Dubbo Hospital
◯ Dunedoo Multi Purpose Service
◯ Gilgandra Multi Purpose Service
◯ Gulgong Multi Purpose Service
◯ Mudgee Health Service
◯ Narromine Health Service
◯ Nyngan Multi Purpose Service
◯ Trangie Multi Purpose Service
◯ Walgett Multipurpose Service
◯ Warren Multi Purpose Service
◯ Wellington Health Service
◯ Lightning Ridge Multi Purpose Service
◯ Lourdes Hospital Dubbo
◯ Bathurst Base Hospital
◯ Blayney Multi Purpose Service
◯ Canowindra Soldiers Memorial Hospital
◯ Condobolin Health Service
◯ Cowra Health Service
◯ Eugowra Memorial Multipurpose Service
◯ Lachlan Health Service - Forbes
◯ Grenfell Multi Purpose Service
◯ Molong Health Service
◯ Oberon Multi Purpose Service
◯ Orange Health Service
◯ Lachlan Health Service - Parkes
◯ Peak Hill Multipurpose Service
◯ Trundle Multi Purpose Health Service
◯ Rylstone Multi Purpose Service
◯ Tottenham Multipurpose Service
◯ Tullamore Multi Purpose Health Service
◯ Balranald Multi Purpose Service

◯ Wentworth Hospital
◯ Broken Hill Hospital
◯ Wilcannia Multi Purpose Service
◯ Thomas Walker Hospital
◯ Cumberland Hospital
◯ Macquarie Hospital
◯ Coral Tree Family Centre
◯ Morisset Hospital
◯ Hunter New England Mental Health Service
◯ Kenmore Hospital
◯ Western District Health Service [Hamilton]
◯ Western District Health Service [Penshurst]
◯ Colac Area Health
◯ Hesse Rural Health Service [Winchelsea]
◯ Otway Health & Community Services [Apollo Bay]
◯ University Hospital Geelong
◯ Casterton Memorial Hospital
◯ Western District Health Service [Coleraine]
◯ South West Healthcare [Warrnambool]
◯ Beaufort & Skipton Health Service [Skipton]
◯ Heywood Rural Health
◯ Timboon & District Healthcare Service
◯ Moyne Health Services [Port Fairy]
◯ Portland District Health
◯ Lorne Community Hospital
◯ The McKellar Centre
◯ South West Healthcare [Camperdown]
◯ Terang & Mortlake Health Service [Terang]
◯ East Grampians Health Service [Ararat]
◯ East Grampians Health Service [Willaura]
◯ Rural Northwest Health [Warracknabeal]
◯ Rural Northwest Health [Hopetoun]
◯ Ballarat Health Services [Base Hospital]
◯ Ballarat Health Services [Queen Elizabeth Campus]
◯ Wimmera Base Hospital [Horsham]
◯ West Wimmera Health Service [Rupanyup]
◯ Stawell Regional Health
◯ West Wimmera Health Service [Nhill]
◯ West Wimmera Health Service [Kaniva]
◯ West Wimmera Health Service [Jeparit]
◯ West Wimmera Health Service [Rainbow]
◯ East Wimmera Health Service [St Arnaud]
◯ Djerriwarrh Health Service [Bacchus Marsh]
◯ Beaufort & Skipton Health Service [Beaufort]
◯ Dimboola District Hospital
◯ Edenhope and District Memorial Hospital
◯ Melton Health
◯ Daylesford District Hospital
◯ Creswick District Hospital
◯ The Bendigo Hospital
◯ Bendigo Health Care Group [Anne Caudle]
◯ Goulburn Valley Health [Waranga]
◯ Kyabram & District Health Service
◯ Kyneton District Health Service
◯ Heathcote Health
◯ Maryborough District Health Service [Maryborough]
◯ Maryborough District Health Service [Dunolly]
◯ Swan Hill District Health [Swan Hill]
◯ Swan Hill District Health [Nyah]
◯ East Wimmera Health Service [Wycheproof]
◯ Cohuna District Hospital
◯ Echuca Regional Health
◯ Kerang District Health
◯ Mildura Base Hospital
◯ Mallee Track Health & Community Service [Ouyen]
◯ Maldon Hospital
◯ Manangatang & District Hospital
◯ Boort District Health
◯ Robinvale District Health Services
◯ Rochester & Elmore District Health Service
◯ East Wimmera Health Service [Donald]

◯ Inglewood & District Health Service
◯ East Wimmera Health Service [Birchip]
◯ East Wimmera Health Service [Charlton]
◯ Castlemaine Health
◯ Goulburn Valley Health [Shepparton]
◯ Goulburn Valley Health [Tatura]
◯ Northeast Health Wangaratta
◯ Tallangatta Health Service
◯ Albury Wodonga Health [Wodonga Campus]
◯ Yarrawonga Health
◯ Alpine Health [Myrtleford]
◯ Alpine Health [Bright]
◯ Alpine Health [Mount Beauty]
◯ Corryong Health
◯ Seymour District Memorial Hospital
◯ Mansfield District Hospital
◯ Alexandra District Hospital
◯ Numurkah & District Health Service
◯ The Kilmore & District Hospital
◯ Yea & District Memorial Hospital
◯ Nathalia District Hospital
◯ Benalla Health
◯ Beechworth Health Service
◯ Cobram District Health
◯ Bairnsdale Regional Health Service
◯ West Gippsland Healthcare Group [Warragul]
◯ Bass Coast Health
◯ Yarram & District Health Service
◯ Omeo District Health
◯ Central Gippsland Health Service [Sale]
◯ Gippsland Southern Health Service - Korumburra
◯ Gippsland Southern Health Service - Leongatha
◯ Latrobe Regional Hospital [Traralgon]
◯ Central Gippsland Health Service [Maffra]
◯ South Gippsland Hospital [Foster]
◯ Orbost Regional Health
◯ Box Hill Hospital
◯ Monash Medical Centre [Clayton]
◯ Maroondah Hospital [East Ringwood]
◯ Angliss Hospital
◯ St George's Health Service- Aged Care
◯ Caritas Christi Hospice [Kew]
◯ Mercy Health - O'Connell Family Centre
◯ The Peter James Centre [East Burwood]
◯ Wantirna Health
◯ Royal Talbot Rehabilitation Centre [Kew]
◯ Healesville Hospital and Yarra Valley Health
◯ Yarra Ranges Health
◯ Monash Medical Centre [Moorabbin]
◯ Rosebud Hospital
◯ Sandringham Hospital
◯ Caulfield Hospital
◯ Kooweerup Regional Health Service
◯ Kingston Centre [Cheltenham]
◯ Dandenong Hospital
◯ Cranbourne Integrated Care Centre
◯ Frankston Hospital
◯ Calvary Health Care Bethlehem
◯ Queen Elizabeth Centre [Noble Park]
◯ Casey Hospital
◯ Mount Eliza Aged Care & Rehabilitation Service
◯ Rosebud Rehabilitation Unit
◯ Golf Links Road Rehabilitation Centre
◯ The Mornington Centre
◯ The Alfred
◯ Austin Hospital [Heidelberg]
◯ Heidelberg Repatriation Hospital [Heidelberg West]
◯ Bundoora Extended Care Centre
◯ Mercy Hospital for Women
◯ Western Hospital [Footscray]
◯ Royal Children's Hospital [Parkville]

◯ Royal Children's Hospital [Travancore Psych]
◯ Royal Women's Hospital [Parkville]
◯ The Royal Victorian Eye & Ear Hospital
◯ The Northern Hospital [Epping]
◯ Werribee Mercy Hospital
◯ Orygen Inpatient Unit
◯ Royal Melbourne Hospital [City Campus]
◯ Royal Melbourne Hospital [Royal Park Campus]
◯ Sunshine Hospital
◯ St Vincent's Hospital [Melbourne]
◯ Williamstown Hospital
◯ Peter MacCallum Cancer Centre
◯ Tweddle Child & Family Health Centre [Footscray]
◯ Sunbury Day Hospital
◯ Broadmeadows Health Service
◯ Craigieburn Health Service
◯ The Royal Dental Hospital of Melbourne
◯ Albury Wodonga Health [Albury Campus]
◯ Victorian Institute of Forensic Mental Health
◯ Victorian Institute of Forensic Mental Health Prison Health Service
◯ Mater Adult Hospital
◯ Mater Children's Hospital
◯ Mater Mothers' Hospital
◯ The Prince Charles Hospital
◯ Royal Children's Hospital [Queensland]
◯ Princess Alexandra Hospital
◯ Ipswich Hospital
◯ Redcliffe Hospital
◯ Ellen Barron Family Centre
◯ Queen Elizabeth II Jubilee Hospital
◯ Wynnum Health Service
◯ Redland Hospital
◯ Logan Hospital
◯ Caboolture Hospital
◯ Beaudesert Hospital
◯ Boonah Hospital
◯ Caloundra Hospital
◯ Esk Hospital
◯ Gatton Hospital
◯ Kilcoy Hospital
◯ Laidley Hospital
◯ Maleny Hospital
◯ Nambour General Hospital
◯ Gold Coast University Hospital
◯ Biggenden Hospital
◯ Bundaberg Base Hospital
◯ Cherbourg Hospital
◯ Childers Hospital
◯ Eidsvold Hospital
◯ Gayndah Hospital
◯ Gin Gin Hospital
◯ Gympie Hospital
◯ Hervey Bay Hospital
◯ Kingaroy Hospital
◯ Maryborough Hospital
◯ Monto Hospital
◯ Mundubbera Hospital
◯ Murgon Hospital
◯ Nanango Hospital
◯ Wondai Hospital
◯ Chinchilla Hospital
◯ Dalby Hospital
◯ Goondiwindi Hospital
◯ Inglewood Hospital
◯ Jandowae Hospital
◯ Miles Hospital
◯ Millmerran Hospital
◯ Oakey Hospital
◯ Stanthorpe Hospital
◯ Tara Hospital

◯ Taroom Hospital
◯ Texas Hospital
◯ Toowoomba Hospital
◯ Warwick Hospital
◯ Augathella Hospital
◯ Charleville Hospital
◯ Cunnamulla Hospital
◯ Dirranbandi Hospital
◯ Injune Hospital
◯ Mitchell Hospital
◯ Mungindi Hospital
◯ Quilpie Hospital
◯ Roma Hospital
◯ St George Hospital Qld
◯ Surat Hospital
◯ Alpha Hospital
◯ Baralaba Hospital
◯ Biloela Hospital
◯ Blackwater Hospital
◯ Emerald Hospital
◯ Gladstone Hospital
◯ Mount Morgan Hospital
◯ Moura Hospital
◯ Rockhampton Hospital
◯ Springsure Hospital
◯ Theodore Hospital
◯ Capricorn Coast Hospital
◯ Woorabinda Hospital
◯ Barcaldine Hospital
◯ Blackall Hospital
◯ Longreach Hospital
◯ Winton Hospital
◯ Clermont Hospital
◯ Mackay Base Hospital
◯ Moranbah Hospital
◯ Proserpine Hospital
◯ Sarina Hospital
◯ Dysart Hospital
◯ Ayr Hospital
◯ Bowen Hospital
◯ Charters Towers Hospital
◯ Collinsville Hospital
◯ Home Hill Hospital
◯ Ingham Hospital
◯ Joyce Palmer Health Service
◯ Townsville University Hospital
◯ Royal Brisbane & Women's Hospital
◯ Atherton Hospital
◯ Babinda Hospital
◯ Bamaga Hospital
◯ Cairns Hospital
◯ Cooktown Hospital
◯ Gordonvale Hospital
◯ Herberton Hospital
◯ Innisfail Hospital
◯ Mareeba Hospital
◯ Mossman Hospital
◯ Thursday Island Hospital
◯ Tully Hospital
◯ Weipa Hospital
◯ Cloncurry Hospital
◯ Hughenden Hospital
◯ Julia Creek Hospital
◯ Mount Isa Base Hospital
◯ Normanton Hospital
◯ Richmond Hospital
◯ Mornington Island Hospital
◯ Doomadgee Hospital
◯ Baillie Henderson Hospital
◯ Charters Towers Rehabilitation Unit
◯ Kirwan Rehabilitation Unit

◯ The Park Centre For Mental Health
◯ Hampstead Rehabilitation Centre
◯ The Queen Elizabeth Hospital
◯ Royal Adelaide Hospital
◯ Lyell McEwin Hospital
◯ Modbury Hospital
◯ Pregnancy Advisory Centre
◯ St Margaret's Hospital
◯ Women's and Children's Hospital
◯ Wudinna Hospital
◯ Cleve District Hospital and Aged Care
◯ Cowell District Hospital and Aged Care
◯ Cummins and District Memorial Hospital
◯ Elliston Hospital
◯ Kimba District Hospital and Aged Care
◯ Ceduna District Health Service
◯ Port Lincoln Health Service
◯ Streaky Bay Hospital
◯ Tumby Bay Hospital and Health Services
◯ Gumeracha District Soldiers' Memorial Hospital
◯ Kangaroo Island Health Service
◯ Karoonda and District Soldiers' Memorial Hospital
◯ Lameroo District Health Service
◯ Tailem Bend District Hospital
◯ Mannum District Hospital
◯ Meningie and Districts Memorial Hospital and Health Services
◯ Mount Barker District Soldiers' Memorial Hospital
◯ Mount Pleasant District Hospital
◯ Murray Bridge Soldiers' Memorial Hospital
◯ Pinnaroo Soldiers' Memorial Hospital
◯ South Coast District Hospital
◯ Strathalbyn and District Health Service
◯ Booleroo Centre District Hospital and Health Services
◯ Crystal Brook and District Hospital
◯ Jamestown Hospital and Health Service
◯ Laura and District Hospital
◯ Orroroo and District Health Service
◯ Peterborough Soldiers' Memorial Hospital
◯ Port Broughton and District Hospital and Health Service
◯ Port Pirie Regional Health Service
◯ McLaren Vale & Districts War Memorial Hospital Inc
◯ Coober Pedy Hospital and Health Service
◯ Hawker Memorial Hospital
◯ Leigh Creek Health Service
◯ Port Augusta Hospital and Regional Health Services
◯ Quorn Health Service
◯ Roxby Downs Health Service
◯ Whyalla Hospital and Health Services
◯ Barmera Health Service
◯ Riverland General Hospital
◯ Loxton Hospital Complex
◯ Renmark Paringa District Hospital
◯ Waikerie Health Service
◯ Bordertown Memorial Hospital
◯ Kingston Soldiers' Memorial Hospital
◯ Millicent and District Hospital and Health Service
◯ Mount Gambier and Districts Health Service
◯ Naracoorte Health Service
◯ Penola War Memorial Hospital
◯ Flinders Medical Centre
◯ Repatriation General Hospital
◯ Noarlunga Hospital
◯ Gawler Health Service
◯ Angaston District Hospital
◯ Balaklava Soldiers' Memorial District Hospital
◯ Burra Hospital
◯ Clare Hospital
◯ Eudunda Hospital

◯ Kapunda Hospital
◯ Central Yorke Peninsula Hospital (Maitland)
◯ Riverton District Soldiers' Memorial Hospital
◯ Snowtown Hospital and Health Service
◯ Southern Yorke Peninsula Health Service (Yorketown)
◯ Tanunda War Memorial Hospital
◯ Northern Yorke Peninsula Health Service (Wallaroo)
◯ Oakden Hospital
◯ Glenside Health Services
◯ Princess Margaret Hospital for Children
◯ King Edward Memorial Hospital
◯ Sir Charles Gairdner Hospital
◯ Selby Authorised Lodge
◯ Osborne Park Hospital
◯ Swan District Hospital
◯ Kalamunda Hospital
◯ Next Step Drug And Alcohol Services, East Perth
◯ Joondalup Health Campus (Public)
◯ Royal Perth Hospital Shenton Park Campus
◯ Royal Perth Hospital Wellington Street Campus
◯ Fremantle Hospital and Health Service
◯ Kaleeya Hospital
◯ Armadale-Kelmscott Memorial Hospital
◯ Bentley Health Service
◯ Rockingham General Hospital
◯ Murray District Hospital
◯ Peel Health Campus
◯ Fitzroy Crossing Hospital
◯ Halls Creek Hospital
◯ Broome Hospital
◯ Derby Hospital
◯ Wyndham Hospital
◯ Kununurra Hospital
◯ Hedland Health Campus
◯ Onslow Hospital
◯ Roebourne Hospital
◯ Tom Price Hospital
◯ Newman Hospital
◯ Paraburdoo Hospital
◯ Nickol Bay Hospital
◯ Carnarvon Hospital
◯ Exmouth Hospital
◯ Geraldton Hospital
◯ Meekatharra Hospital
◯ Morawa Health Service
◯ Mullewa Health Service
◯ Northampton Kalbarri Health Service
◯ North Midlands Health Service
◯ Kalbarri Health Centre
◯ Dongara Eneabba Mingenew Health Service
◯ Lake Grace Hospital
◯ Merredin Hospital
◯ Narrogin Hospital
◯ Northam Hospital
◯ Wagin Hospital
◯ York Hospital
◯ Beverley Hospital
◯ Boddington Hospital
◯ Bruce Rock Memorial Hospital
◯ Corrigin Hospital
◯ Cunderdin Hospital
◯ Dalwallinu Hospital
◯ Dumbleyung Memorial Hospital
◯ Kellerberrin Memorial Hospital
◯ Goomalling Hospital
◯ Kondinin Hospital
◯ Kununoppin Health Service
◯ Moora Hospital
◯ Narembeen Memorial Hospital
◯ Pingelly Health Centre
◯ Southern Cross Hospital

◯ Wongan Hills Hospital
◯ Wyalkatchem-Koorda and Districts Hospital
◯ Quairading Hospital
◯ Esperance Hospital
◯ Kalgoorlie Health Campus
◯ Laverton Hospital
◯ Leonora Hospital
◯ Norseman Hospital
◯ Albany Hospital
◯ Denmark Hospital and Health Service
◯ Katanning Hospital
◯ Gnowangerup Hospital
◯ Plantagenet Hospital
◯ Ravensthorpe Health Centre
◯ Kojonup Hospital
◯ Augusta Hospital
◯ Bunbury Hospital
◯ Busselton Health Campus
◯ Collie Hospital
◯ Margaret River Hospital
◯ Donnybrook Hospital
◯ Harvey Hospital
◯ Nannup Hospital
◯ Pemberton Hospital
◯ Boyup Brook Soldiers Memorial Hospital
◯ Warren Hospital
◯ Bridgetown Hospital
◯ Graylands Selby-Lemnos and Special Care Health Service
◯ Royal Hobart Hospital
◯ Launceston General Hospital
◯ North West Regional Hospital
◯ Mersey Community Hospital
◯ Royal Darwin Hospital
◯ Alice Springs Hospital
◯ Tennant Creek Hospital
◯ Katherine Hospital
◯ Gove Hospital
◯ The Canberra Hospital
◯ Calvary Public Hospital Bruce
◯ Robina Hospital
◯ Queensland Children's Hospital
◯ Fiona Stanley Hospital
◯ St John of God Midland Public Hospital
◯ Byron Central Hospital
◯ Women's at Sandringham
◯ Sunshine Coast University Hospital
◯ Perth Children's Hospital
◯ Karratha Health Campus
◯ Northern Beaches Hospital
◯ Palmerston Regional Hospital
◯ Careplans Assessment Victoria
◯ Cudal Health Service
◯ Dame Phyllis Frost Centre - Marmak unit
◯ Goodooga Health Service
◯ Ivanhoe Hospital
◯ Menindee Health Service
◯ QEII Family Centre
◯ Sydney Dental Hospital
◯ Sydney Road Clinic
◯ Tibooburra Health Service
◯ Mallee Track Health & Community Service [Sea Lake]
◯ Ursula Frayne Centre
◯ Surgical Treatment and Rehabilitation Service

Please add the name of your hospital here	______________________
How many rheumatologists work in your department?	◯ 0 ◯ 1 ◯ 2 ◯ 3<br>◯ 4 ◯ 5 ◯ 6 ◯ 7<br>◯ 8 ◯ 9 ◯ 10+
What is the total full-time equivalent of rheumatologists in your department? For example, 4 rheumatologists working full-time would be 4.0.	______________________
Do you have Visiting Medical Officers in your department?	◯ Yes ◯ No
What is the total full-time equivalent of visiting medical officers in your department? For example, 4 visiting medical officers working full-time would be 4.0.	______________________

Nurse

Do you have a nurse or nurse practitioner working WITHIN your rheumatology department?	◯ Yes ◯ No
What prevents you from having nurse/s working WITHIN your rheumatology department (choose all that apply).	☐ Funding<br>☐ Lack of clinical need<br>☐ Lack of time/motivation to create a position<br>☐ Difficulty finding health professionals with sufficient knowledge/skills<br>☐ Access to health professionals OUTSIDE the department with sufficient skills/knowledge<br>☐ Limited space/facilities for extra health professionals WITHIN the department<br>☐ Other
Please describe the other reason.	__________________________________
Please specify the type of nurse/s working WITHIN your rheumatology department. You can select more than 1 type of nurse.	☐ Nurse ☐ Nurse Practitioner
How many nurses do you have working WITHIN your rheumatology department in total? Please select.	◯ 1 ◯ 2 ◯ 3 ◯ 4+
What is the full time equivalent (FTE) of the nurse/s? Please write as an equivalent i.e. 1 day per week would be 0.2 FTE.	__________________________________
Is the position for the nurse/s funded by your organisation?	◯ Yes ◯ No
Please specify the funding source for this position.	__________________________________
Do you have a nurse or nurse practitioner working OUTSIDE your rheumatology department but within your organisation to whom staff routinely refer?	◯ Yes ◯ No
What prevents you from referring to nurse/s working OUTSIDE your rheumatology department (choose all that apply).	☐ There is a nurse WITHIN the rheumatology department<br>☐ Funding<br>☐ Lack of sufficient knowledge/skills<br>☐ No referral pathway within the organisation<br>☐ Lack of clinical need<br>☐ The health professional is too busy to see our patients<br>☐ Other
Please describe the other reason.	__________________________________
Is the position for the nurse/s funded by your organisation?	◯ Yes ◯ No
Please specify the funding source for this position.	__________________________________

Do nurses contribute to specialised services for your patients (e.g. scleroderma clinic)?	◯ Yes<br>◯ No
Please describe the specialised services to which nurses contribute?	__________________________________
What do you think stops nurses contributing to specialised services?	__________________________________

Physiotherapist

Do you have physiotherapists working WITHIN your rheumatology department?	◯ Yes ◯ No
What prevents you from having physiotherapist/s working WITHIN your rheumatology department (choose all that apply).	☐ Funding<br>☐ Lack of clinical need<br>☐ Lack of time/motivation to create a position<br>☐ Difficulty finding health professionals with sufficient knowledge/skills<br>☐ Access to health professionals OUTSIDE the department with sufficient skills/knowledge<br>☐ Limited space/facilities for extra health professionals WITHIN the department<br>☐ Other
Please describe the other reason.	__________________________________
How many physiotherapists do you have working WITHIN your rheumatology department?	◯ 1 ◯ 2 ◯ 3 ◯ 4+
What is the full time equivalent (FTE) of the physiotherapist/s? Please write as an equivalent i.e. 1 day per week would be 0.2 FTE.	____________________________
Is the position for the physiotherapist/s funded by your organisation?	◯ Yes ◯ No
Please specify the funding source for this position:	____________________________
Do you have physiotherapist/s working OUTSIDE your rheumatology department but within your organisation to whom staff in your department routinely refer?	◯ Yes ◯ No
What prevents you from referring to physiotherapist/s working OUTSIDE your rheumatology department (choose all that apply).	☐ There is a physiotherapist WITHIN the rheumatology department<br>☐ Funding<br>☐ Lack of sufficient knowledge/skills<br>☐ No referral pathway within the organisation<br>☐ Lack of clinical need<br>☐ The health professional is too busy to see our patients<br>☐ Other
Please describe the other reason.	____________________________
Is the position for the physiotherapist/s funded by your organisation?	◯ Yes ◯ No
Please specify the funding source for this position:	____________________________
Do physiotherapists contribute to specialised services for your patients (e.g. hand therapy)?	◯ Yes<br>◯ No

Please describe the specialised services to which physiotherapists contribute

____

What do you think stops physiotherapists contributing to specialised services?

____

Podiatrist

Do you have a podiatrist working WITHIN your rheumatology department?	◯ Yes ◯ No
What prevents you from having podiatrist/s working WITHIN your rheumatology department (choose all that apply).	☐ Funding<br>☐ Lack of clinical need<br>☐ Lack of time/motivation to create a position<br>☐ Difficulty finding health professionals with sufficient knowledge/skills<br>☐ Access to health professionals OUTSIDE the department with sufficient skills/knowledge<br>☐ Limited space/facilities for extra health professionals WITHIN the department<br>☐ Other
Please describe the other reason.	__________________________________
How many podiatrists do you have working WITHIN your rheumatology department in total?	◯ 1 ◯ 2 ◯ 3 ◯ 4+
What is the full time equivalent (FTE) of the podiatrist/s? Please write as an equivalent i.e. 1 day per week would be 0.2 FTE.	__________________________________
Is the position for the podiatrist/s funded by your organisation?	◯ Yes ◯ No
Please specify the funding source for this position.	__________________________________
Do you have a podiatrist working OUTSIDE your rheumatology department but within your organisation to whom staff in your department routinely refer?	◯ Yes ◯ No
What prevents you from referring to podiatrist/s working OUTSIDE your rheumatology department (choose all that apply).	☐ There is a podiatrist WITHIN the rheumatology department<br>☐ Funding<br>☐ Lack of sufficient knowledge/skills<br>☐ No referral pathway within the organisation<br>☐ Lack of clinical need<br>☐ The health professional is too busy to see our patients<br>☐ Other
Please describe the other reason.	__________________________________
Is the position for the podiatrist/s funded by your organisation?	◯ Yes ◯ No
Please specify the funding source for this position.	__________________________________
Do podiatrists contribute to specialised services for your patients (e.g. rheumatoid arthritis clinic)?	◯ Yes<br>◯ No

Please describe the specialised services to which podiatrists contribute

____

What do you think stops podiatrists contributing to specialised services?

____

Psychologist

Do you have psychologist/s working WITHIN your rheumatology department?	◯ Yes ◯ No
What prevents you from having psychologist/s working WITHIN your rheumatology department (choose all that apply).	☐ Funding<br>☐ Lack of clinical need<br>☐ Lack of time/motivation to create a position<br>☐ Difficulty finding health professionals with sufficient knowledge/skills<br>☐ Access to health professionals OUTSIDE the department with sufficient skills/knowledge<br>☐ Limited space/facilities for extra health professionals WITHIN the department<br>☐ Other
Please describe the other reason.	__________________________
How many psychologists do you have working WITHIN your rheumatology department in total?	◯ 1 ◯ 2 ◯ 3 ◯ 4+
What is the full time equivalent (FTE) of the psychologist/s?<br>Please write as an equivalent i.e. 1 day per week would be 0.2 FTE.	__________________________
Is the position for the psychologist/s funded by your organisation?	◯ Yes ◯ No
Please specify the funding source for this position.	__________________________
Do you have psychologist/s working OUTSIDE your rheumatology department but within your organisation to whom staff in your department routinely refer?	◯ Yes ◯ No
What prevents you from referring to psychologist/s working OUTSIDE your rheumatology department (choose all that apply).	☐ There is a psychologist WITHIN the rheumatology department<br>☐ Funding<br>☐ Lack of sufficient knowledge/skills<br>☐ No referral pathway within the organisation<br>☐ Lack of clinical need<br>☐ The health professional is too busy to see our patients<br>☐ Other
Please describe the other reason.	__________________________
Is the position for the psychologist/s funded by your organisation?	◯ Yes ◯ No
Please specify the funding source for this position.	__________________________
Do psychologists contribute to specialised services for your patients?	◯ Yes<br>◯ No

Please describe the specialised services to which psychologists contribute

____

What do you think stops psychologists contributing to specialised services?

____

Occupational therapist

Do you have an occupational therapist/s working WITHIN your rheumatology department?	◯ Yes ◯ No
What prevents you from having occupational therapist/s working WITHIN your rheumatology department (choose all that apply).	☐ Funding<br>☐ Lack of clinical need<br>☐ Lack of time/motivation to create a position<br>☐ Difficulty finding health professionals with sufficient knowledge/skills<br>☐ Access to health professionals OUTSIDE the department with sufficient skills/knowledge<br>☐ Limited space/facilities for extra health professionals WITHIN the department<br>☐ Other
Please describe the other reason.	__________________________________
How many occupational therapists do you have working WITHIN your rheumatology department in total?	◯ 1 ◯ 2 ◯ 3 ◯ 4+
What is the full time equivalent (FTE) of the occupational therapist/s?<br>Please write as an equivalent i.e. 1 day per week would be 0.2 FTE.	__________________________________
Is the position for the occupational therapist/s funded by your organisation?	◯ Yes ◯ No
Please specify the funding source for this position.	__________________________________
Do you have an occupational therapist/s working OUTSIDE your rheumatology department but within your organisation to whom staff in your department routinely refer?	◯ Yes ◯ No
What prevents you from referring to occupational therapist/s working OUTSIDE your rheumatology department (choose all that apply).	☐ There is an occupational therapist WITHIN the rheumatology department<br>☐ Funding<br>☐ Lack of sufficient knowledge/skills<br>☐ No referral pathway within the organisation<br>☐ Lack of clinical need<br>☐ The health professional is too busy to see our patients<br>☐ Other
Please describe the other reason.	__________________________________
Is the position for the occupational therapist/s funded by your organisation?	◯ Yes ◯ No
Please specify the funding source for this position.	__________________________________
Do occupational therapists contribute to specialised services for your patients (e.g. hand therapy)?	◯ Yes<br>◯ No

Please describe the specialised services to which occupational therapists contribute

____

What do you think stops occupational therapists contributing to specialised services?

____

Pharmacist

Do you have a pharmacist working WITHIN your rheumatology department?	◯ Yes ◯ No
What prevents you from having pharmacist/s working WITHIN your rheumatology department (choose all that apply).	☐ Funding<br>☐ Lack of clinical need<br>☐ Lack of time/motivation to create a position<br>☐ Difficulty finding health professionals with sufficient knowledge/skills<br>☐ Access to health professionals OUTSIDE the department with sufficient skills/knowledge<br>☐ Limited space/facilities for extra health professionals WITHIN the department<br>☐ Other
Please describe the other reason	__________________________________
How many pharmacists do you have working WITHIN your rheumatology department?	◯ 1 ◯ 2 ◯ 3+
What is the full time equivalent (FTE) of the pharmacist/s?<br>Please write as an equivalent i.e. 1 day per week would be 0.2 FTE.	__________________________________
Is the position for the pharmacist/s funded by your organisation?	◯ Yes ◯ No
Please specify the funding source for this position.	__________________________________
Do pharmacists contribute to specialised services for your patients (e.g. inflammatory bowel disease clinics)?	◯ Yes<br>◯ No
Please describe the specialised services to which pharmacists contribute	__________________________________
What do you think stops pharmacists from contributing to specialist services?	__________________________________

Paedriatric services

Does your department offer services to paediatric patients?	◯ Yes<br>◯ No
Do nursing and/or allied health staff work with paediatric patients WITHIN your department?	◯ Yes<br>◯ No
What prevents you from having nursing/allied health staff working with paediatric patients WITHIN your rheumatology department (choose all that apply).	☐ Funding<br>☐ Lack of clinical need<br>☐ Lack of time/motivation to create a position<br>☐ Difficulty finding health professionals with sufficient knowledge/skills<br>☐ Access to health professionals OUTSIDE the department with sufficient skills/knowledge<br>☐ Limited space/facilities for extra health professionals WITHIN the department<br>☐ Other
Please describe the other reason	__________________________________
Choose the nursing and/or allied health staff who work with paediatric patients (select all that apply).	☐ Nurse<br>☐ Nurse practitioner<br>☐ Physiotherapist<br>☐ Podiatrist<br>☐ Occupational therapist<br>☐ Psychologist<br>☐ Pharmacist<br>☐ Other
What is the FTE of the nurse/s in this role?	__________________________________
What is the FTE of the nurse practitioner/s in this role?	__________________________________
What is the FTE of the physiotherapist/s in this role?	__________________________________
What is the FTE of the podiatrist/s in this role?	__________________________________
What is the FTE of the occupational therapist/s in this role?	__________________________________
What is the FTE of the psychologist/s in this role?	__________________________________
What is the FTE of the pharmacist/s in this role?	__________________________________
What is the FTE of the other health professional/s in this role?	__________________________________

Do you have nursing or allied health professionals OUTSIDE your rheumatology department but within your organisation to whom staff in your department routinely refer?	◯ Yes ◯ No
What prevents you from referring to allied health professionals working with paediatric patients OUTSIDE your rheumatology department (choose all that apply).	☐ There is are allied health professionals WITHIN the rheumatology department working with paediatric patients<br>☐ Funding<br>☐ Lack of sufficient knowledge/skills<br>☐ No referral pathway within the organisation<br>☐ Lack of clinical need<br>☐ The health professional is too busy to see our patients<br>☐ Other
Please describe the other reason	__________________________________
Choose the nursing and/or allied health staff who work with paediatric patients (select all that apply).	☐ Nurse<br>☐ Nurse practitioner<br>☐ Physiotherapist<br>☐ Podiatrist<br>☐ Occupational therapist<br>☐ Psychologist<br>☐ Pharmacist<br>☐ Other
Please describe the other health professional	__________________________________
